# Distinctive location of piscine intestinal coccidiosis in Asian seabass fingerlings

**DOI:** 10.14202/vetworld.2022.2164-2171

**Published:** 2022-09-09

**Authors:** Watcharapol Suyapoh, Peerapon Sornying, Chanoknun Thanomsub, Khemjira Kraonual, Korsin Jantana, Sirikachorn Tangkawattana

**Affiliations:** 1 Department of Veterinary Science, Faculty of Veterinary Science, Prince of Songkla University, Songkhla, Thailand; 2 WHO Collaborating Centre for Research and Control of Opisthorchiasis (Southeast Asian Liver Fluke Disease), Tropical Diseases Research Center, Faculty of Medicine, Khon Kaen University, Khon Kaen, Thailand; 3 Faculty of Veterinary Medicine, Khon Kaen University, Khon Kaen, Thailand

**Keywords:** Asian seabass, coccidian, fingerling, histopathology, *Lates calcarifer*, small intestine

## Abstract

**Background and Aim:**

Coccidian infection (coccidiosis) is one of the most important causes of illness and death in the fish population, including Asian sea bass. The fingerling developmental stage is sensitive to various infectious agents. Economic losses are sustained by the sea bass aquaculture industry due to coccidiosis annually. However, the related pathological changes in the Asian sea bass fingerlings’ three-part intestine remain unknown. This study aimed to investigate the Asian sea bass fingerlings’ infection rate, infection location and site, and specific pathological lesions in the small intestinal tissues in a marine cage farming operation.

**Materials and Methods:**

A cross-sectional study was conducted on 44 fingerling fishes. Major coccidia proportions were identified morphologically at both the macroscopic and microscopic levels. The infection number was determined based on coccidia presence at various intestinal locations and sites. All areas were assessed for pathological lesions using semi-quantitative grading. Analysis of variance was used to perform all data analyses using the SPSS software. Data were expressed as means ± standard deviation. p < 0.05 was considered statistically significant.

**Results:**

All Asian sea bass fingerlings studied were infected with coccidia. Enteritis and mucosal necrosis were distinct lesions found in the anterior intestine, which had the highest infection rate (49.94%), followed by the mid intestine (35.63%), and the posterior intestine (22.43%). The most common coccidian infection site was extracellular (subepithelial), followed by intracytoplasmic, and epicellular sites. Histopathological lesion determination revealed that intestinal tissue inflammation and epithelial injuries were predominantly seen in the anterior gut (p < 0.05).

**Conclusion:**

There was a high coccidian infection rate in Asian sea bass fingerlings from marine cage farming operations. Infection and intestinal damage at the anterior intestine, a major site, led to fingerling death. Disease prevention in the nursery should be intensive from the fingerling period to decrease the fatality rate caused by coccidia.

## Introduction

The Asian sea bass or barramundi (*Lates calcarifer*) is a staple in aquaculture. It has been increasing in economic value in the Indo–Pacific region, especially in Southeast Asia and Australia [1, 2]. The species’ production has seen a 41% growth, reaching 178 million tons in the 2000–2019 period and is included in the world fishery and aquaculture manufacturing [3]. Major economic losses from infectious diseases that cause high mortality in the farming system, such as infectious diseases caused by viruses [4–6], Streptococci [7], parasitic gill copepods [8], and monogenean parasites [9, 10], have been reported intensively due to the high economic importance of Asian sea bass aquaculture. High mortality commonly occurs in fries and fingerlings [11]. In wild fish, most piscine coccidiosis infections are asymptomatic, but a recent report showed severe pathological lesions and mortality under nursery culture conditions in Asian sea bass fries infected with coccidia [2]. However, the coccidiosis effects on the fingerling stage have not yet been elucidated.

Intestinal coccidiosis is a disease caused by the common piscine apicomplexan parasite genera, including *Eimeria* spp., *Goussia* spp., and *Cryptosporidium* spp. [2, 12–14]. The pathogenicity of this disease depends on individual fish sensitivity, inflammatory responses, the enterocytes turnover rate, bacterial co-infection, low daily water exchange rates, and intestinal location [15, 16]. Infections are associated with pathological changes in the intestinal tissues in the high-pathogenicity response in many fish species, such as denuded intestinal epithelium, intense inflammation, focal necrosis, intestinal epithelium, and mucoid casts containing parasite stages [2, 16, 17, 18].

A cross-sectional study was designed to determine the intestinal coccidia infection rate, the intestinal location and site of infection, and the histopathological abnormalities associated with coccidiosis in Asian sea bass fingerlings to gain more insight into intestinal pathological development. Data from this study will help researchers and fish farmers to understand the tissue injuries associated with intestinal coccidiosis in Asian seabass and lead to a decrease in fingerling loss in aquaculture.

## Materials and Methods

### Ethical approval

All animal management and experimental protocols were approved by the Institutional Animal Care and Use Committee, Prince of Songkla University (MHESI 68014/442).

### Study period and location

The study was conducted during November and December 2021. A cross-sectional study was conducted on 44 Asian sea bass fingerlings from marine cage farming in the Satun province, Thailand.

### Experimental design and sample collection

**Figure-1:**
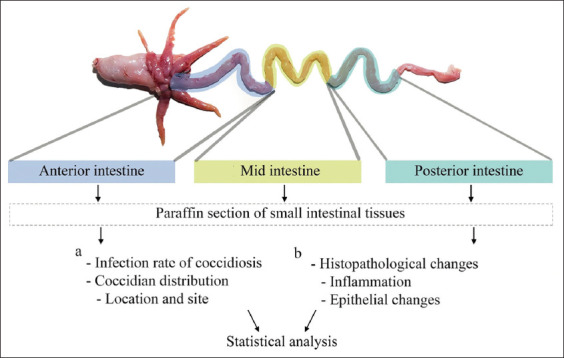
Experimental design. (a) The study of coccidian infection included the infection rate and parasitic distribution with the infection number in 44 Asian sea bass fingerlings. (b) The semi-quantitative evaluation of histopathological changes in the small intestine focused on inflammation and intestinal epithelial changes.

The G*Power software version 3.1 (Informer Technologies, Inc., Germany) was used to calculate the sample size [19]. Fingerlings were transferred to the Department of Veterinary Sciences, Faculty of Veterinary Sciences at the Prince of Songkla University. Fishes were euthanized with eugenol 0.2 mL, as previously described [20]. Briefly, 0.05 ml of clove oil was mixed with 500 ml of dechlorinated water. Individual fish was placed into the container for 30 minutes, then developed a depth of anesthesia. The stomach, small intestines, and large intestines were collected and separated. The small intestine was dissected into three parts: Anterior, mid, and posterior intestine. This followed the criteria of Kathiresan *et al*. [21]. All intestinal samples were fixed in 10% buffered formalin and subsequently processed by routine histological techniques. Hematoxylin and eosin were used to stain the paraffin sections. The coccidian infection rate, parasitic distribution, infection number, and histopathological changes were investigated (Figure-1).

### Coccidia detection and assessment

Nikon advanced upright microscope with a VDO capture digital camera (ECLIPSE Ni-U) (Nikon, Tokyo, Japan) was used for histological examination to perform morphological identification. The parasite and histopathological lesions were investigated from the stomach, three different locations in the small intestine (anterior, mid, and posterior), and the large intestine. The epicellular, intracytoplasmic, and extracellular (subepithelial, submucosal) are the three different epithelial layers observed for coccidia presence. Morphological identification was performed following previous studies with respect to the parasitic stages [17, 22]. A parasitologist confirmed all morphological analyses. Quantitatively, meronts, gamonts, and unsporulated oocysts were counted in five non-overlapping fields of a light microscope. The coccidian infection rate, proportion, and number were determined.

### Semi-quantitative histopathological study

Intestinal submucosal inflammation, intraepithelial lymphocyte infiltration, extension of inflammation, intestinal necrosis, intestinal congestion, and epithelial desquamation were included in the histopathological evaluation. The cell identification criteria were based on Clauss *et al*. [23]. A light microscope (ECLIPSE E200, Nikon, Tokyo, Japan) at 40 × was used to investigate the five non-overlapping microscopic fields in each intestinal location. Semi-quantitative grading was established from previous related research. All severities scored as absent, mild, moderate, and severe were modified and clarified with the quantitative proportion as follows: absent = no lesion development or ≤1% lesion development, mild = 2–25% lesion development, moderate = 26–50% lesion development, and severe = >50% lesion development. The grading details are shown in Table-1 [24-26].

**Table-1:** Semi-quantitative grading of the main histopathological changes in intestinal tissue from Asian sea bass.

Parameter	Grading	Reference
Intestinal inflammation	
Submucosal inflammation	0 = Not present 1 = Mild 2 = Moderate 3 = Severe	[24]
Intraepithelial lymphocyte infiltration	0 = Absent 1 = Mild 2 = Moderate 3 = Severe	[25]
Extension of inflammation	0 = Mucosa 1 = Mucosa and submucosa 2 = Mucosa, submucosa, and sometimes transmural 3 = Mucosa, submucosa, and often transmural	[24]
Intestinal congestion/hyperemia	0 = No 1 = Mild 2 = Moderate 3 = Severe	[26]
Epithelial damage	
Mucosal necrosis	0 = 0–1% 1 = 2–25% 2 = 26–50% 3 = >50%	
Epithelial desquamation	0 = Absent 1 = Mild 2 = Moderate 3 = Severe	[26]

### Statistical analysis

The statistical package for the social sciences (SPSS) version 23.0 (SPSS Inc., USA) was used to statistically analyze all results. Analysis of variance was used to compare data from multiple locations and sites. Results were considered statistically significant at p < 0.05.

## Results

### Coccidia detection and locations of lesions in Asian sea bass fingerlings

**Figure-2:**
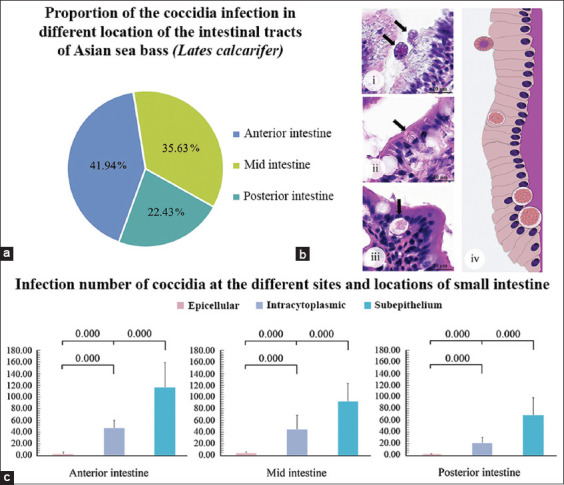
Proportions and infection numbers of coccidia in fingerling fishes. (a) The proportions of meronts, gamonts, and unsporulated oocysts in different locations of the small intestine. (b) Representative micrographs of the main sites of infection: (bi) Epicellular, (bii) intracytoplasmic), (biii) extracellular or subepithelium, (biv) and illustration of all sites of infection. (c) Infection number of coccidia in various sites of the anterior, mid, and posterior locations. Subepithelial coccidia displayed the highest infection rate in all small intestinal locations, and the difference was significant (p < 0.05).

The Asian sea bass fingerling stage is sensitive to infection and loss in aquaculture. The coccidia infection rate was first explored in different small intestinal locations to assess health status related to coccidian infection. All 44 fishes examined were positive for coccidia, that is, a 100% infection rate. No lesions were observed in the stomachs and large intestines of fingerlings. Intestinal lesions and coccidia were present predominantly in the small intestine. The highest parasite proportion (41.94%) was detected in the anterior gut, followed by the mid and posterior gut, with proportions of 35.63% and 22.43%, respectively (Figure-2a). Quantitative detection of the coccidian stages at three sites, including the epicellular, intracytoplasmic, and extracellular or subepithelial layers, was clarified (Figures-2bi–iv). Coccidia presence was significant predominantly in the extracellular or subepithelial sites of all intestinal locations. Infection numbers in detail are shown in Table-2.

**Table-2:** Semi-quantitative inflammatory scores of the intestinal tissues of Asian sea bass fingerlings with natural coccidia infections.

Parameters	Intestinal locations (Mean ± SD)		

	Anterior	Mid	Posterior
Submucosal inflammation	2.30 ± 0.55^Σ^	2.10 ± 0.45^Ω^	1.76 ± 0.47
Intraepithelial lymphocyte infiltration	1.66 ± 0.59^Δ,Σ^	1.35 ± 0.44^Ω^	1.13 ± 0.21
Extension of inflammation	1.26 ± 0.42^Σ^	1.14 ± 0.35	1.06 ± 0.22
Vascular congestion	1.45 ± 0.24^Σ^	1.34 ± 0.31^Ω^	1.17 ± 0.24

Superscript indicates a significant difference between locations at *P<*0.05, i.e., Δ = groups 1–2, Σ = groups 1–3, Ω = groups 2–3 in the same parameter

### Histopathological lesions related to coccidiosis in Asian seabass fingerlings

The three different small intestine locations were further examined because there were no pathological changes in the stomach and large intestine. Major pathological changes found in the small intestine of Asian sea bass fingerlings were categorized into intestinal inflammation and epithelial changes.

### Intestinal inflammation

**Figure-3:**
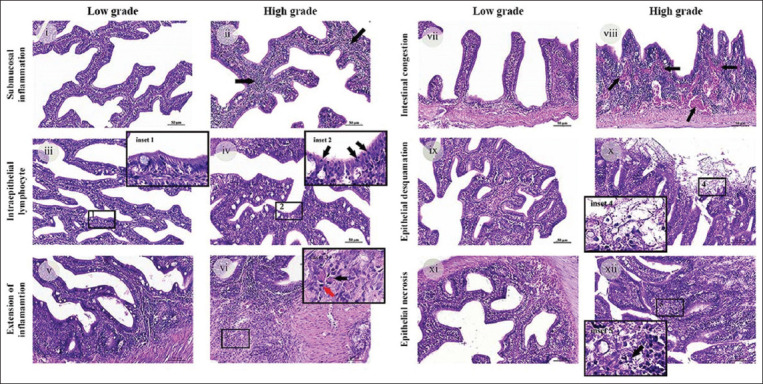
Representative photomicrographs of intestinal inflammation and epithelial alteration. (i and ii) Low and high grade of submucosal inflammation. (iii and iv) Low and high grade of intraepithelial lymphocyte infiltration. The inset illustrates the number of lymphocytes in intestinal epithelial cells of the anterior intestine (inset 2, black arrow). Few such cells were detected in the posterior intestine (inset 1). (v–vi) Low- and high-grade extensive inflammation. (vi) At higher magnification (inset 3), eosinophil infiltration (black arrow) with lymphocytes (red arrow) was observed. (vii and viii) Low- and high-grade vascular congestion. (ix) Normal mucosal surface of the fingerling intestine, (xii) high grade of epithelial desquamation (inset 4, arrow). (xii) The arrow shows focal necrosis of epithelial cells in the anterior gut (inset 5), (xi) compared to unremarkable findings. (i–xii = Hematoxylin and eosin, original magnification, i–xii = 40× magnification, scale bar depicts 50 μm).

**Figure-4:**
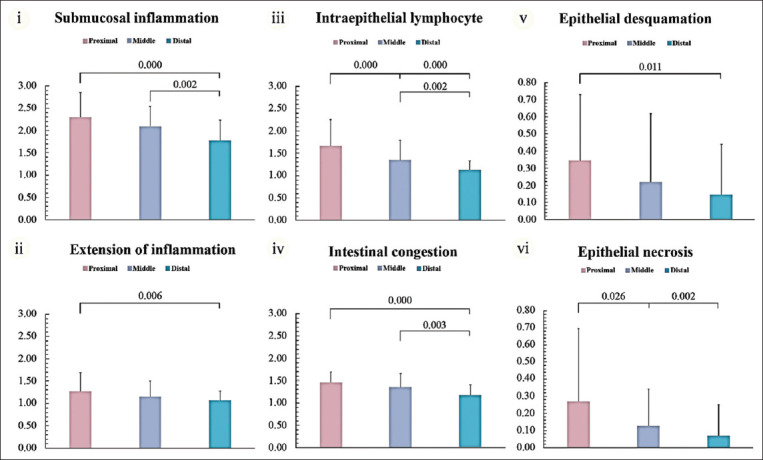
(i–iv) Semi-quantitative comparison of inflammatory and epithelial lesions in the anterior, mid, and posterior intestinal tissues of Asian sea bass fingerlings naturally infected with coccidia. Submucosal inflammation, extensive inflammation, intraepithelial lymphocytes, and intestinal congestion were seen. They were prominent in the anterior intestine and significantly different among these three locations (p < 0.05). (v–vi) Epithelial desquamation and necrosis were distinguished in the anterior intestine and significantly different among locations (p < 0.05).

The inflammatory cell infiltration and vascular congestion that are the outcome of inflammation was further investigated based on the hypothesis that fish coccidia cause significant inflammation in fingerlings. Submucosal inflammation, intraepithelial lymphocyte infiltration, extension of inflammation, and vascular congestion intestinal changes were assessed by histopathological examination of the anterior, mid, and posterior intestine. Inflammation was overall limited to the mucosal and submucosal intestinal layers inhabited by the coccidia stages (Figures-3i–iv). Some fish developed extensive inflammation, which was illustrated by invasive lymphocytes and eosinophils infiltration into the muscular layer and gut wall (Figures-3v and vi). Severe active congestion or hyperemia due to blood vessel dilatation with accumulation of red blood cells at the site of inflammation (Figures-3vii and viii). Lesions in the anterior intestine showed more severe pathological changes than those from the mid and posterior locations. All inflammatory parameters in the anterior intestinal tissues were significantly more progressive than those of the posterior intestine (p < 0.001) (Figures-4i–iv). Moreover, coccidian infection significantly increased the intraepithelial lymphocytes between the anterior and mid locations (p = 0.002) (Figure-4iii). The mid intestine had a greater lymphocyte number infiltrating the submucosa, as well as greater mucosal and vascular congestion than the posterior location (p = 0.002, 0.002, and 0.003, respectively) (Figures-4i, iii, and iv). Semi-quantitative scoring of all inflammatory parameters is detailed in Table-2.

### Epithelial alteration

Mucosal epithelial lesions were investigated, including epithelial desquamation and intestinal necrosis (Figures-3ix–xii). Epithelial desquamation identified by epithelial layer disruption from the basement membrane was observed incidentally in some fishes. Desquamated epithelia were commonly seen in areas with a higher degree of active inflammation and depended on the intestinal location (Figure-3x) in this study. None of these pathological changes were seen in mild-to-moderate degrees of inflammation (Figure-3ix). Epithelial desquamation in the inflamed anterior intestine was more severe than in other locations and significantly higher than in the posterior intestine (p = 0.011) (Figure-4v). No significant difference was detected in epithelial desquamation between the mid gut and other locations.

Disrupted, shrunken, and hypereosinophilic cells with pyknotic, karyorrhectic, or karyolytic nuclei characterized intestinal cellular necrosis. Lesions were detected mainly in severely inflamed intestinal tissue areas, which were abundant around where the coccidia resided (Figure-3xii). The intestinal necrosis degree depended on the location of the infection. Significantly higher tissue necrosis was detected in the anterior gut, compared with the mid and posterior locations (p = 0.026 and 0.002, respectively) (Figure-4vi).

## Discussion

This is the first study that explored the intestinal coccidia present in the Asian sea bass fingerling intestinal tissue in a marine cage farming operation. Meronts, gamonts, and unsporulated oocysts were frequently found at different intestinal tissue sites accompanied by various degrees of histopathological change at three different intestinal locations. Intracellular protozoa are common parasites in fish populations and possible symptomatic illnesses cause [27]. Infection increases fatality and causes economic losses, especially before fish reach maturation [2]. Piscine intestinal coccidia have been reported in both freshwater and marine fish in previous studies [13, 17, 28–30]. This protozoan infection is also detected in the wild and cultured sea bass family, including the European sea bass [31–33] and the Asian sea bass [2, 12]. Here, the highest infection rate of intestinal coccidian was reported at 100% in the fingerling population. Similarly, Lovy and Friend [17] and Gabor *et al*. [34] also reported high rates of various coccidian infections in alewives and juvenile Asian sea bass at 92.00% and 92.5%, respectively. This study found that the highest infection intensity by this parasite was associated with the anterior intestine. This location is also commonly infected by the Goussia species, including *Goussia ameliae*, *Goussia kuehae*, and *Goussia carpelli* [12, 17, 35]; and *Epieimeri*a species, including the *Epieimeria anguillae* [36]. Possible coccidia species infecting the mid to posterior gut include *Goussia alosii* [17] and *Eimeria vanasi* [22]. However, molecular detection should be applied to confirm the identification of coccidian species.

Little is known about the host pattern responses to coccidia in the Asian sea bass fingerling. Inflammation was predominantly seen in small intestinal tissue in this present study. It is widely known that during coccidian infection, mononuclear cells, including lymphocytes, generally infiltrate the submucosal layer throughout the infection period [37–39], followed by intracellular granulocyte and lymphocyte infiltration [39, 40]. A comparative infiltrating inflammatory cell number in different intestinal locations of Asian sea bass fingerlings was reported for the first time in this study. Coccidian infection is confined to the small intestine. Significantly higher lymphocytic inflammation was demonstrated in the anterior gut compared to other intestinal parts. Evidence in juvenile alewives supports these results [17]. The mechanism of leukocyte regulation at specific gut locations was explained by *Enteromyxum* infection. This piscine intracellular parasite regulates T cell populations, especially in the anterior part, whereas dysregulation occurs in the posterior intestine [41]. However, it is still unclear from the available studies how these piscine coccidia are limited to the small intestine.

An association between severe inflammation of the anterior intestine and heavy coccidian infection in fingerling fish was interestingly found. Juvenile fish have a weaker immune system than adults and thus, less protection from parasites [42]. This increases disease susceptibility in younger fish [43]. Furthermore, the anterior intestine plays an important role in immune homeostasis regulation in teleost fish [17]. Alterations of the anterior part commonly cause an immunocompromised stage in young fish and promote heavy coccidia infection [39]. Enterocytes burst out in the host-parasite interaction, releasing the parasitic stages and inducing severe inflammatory responses during parasite excystment [39]. Intestinal congestion is a common lesion associated with inflammation and is generally reported in fish with coccidiosis [17, 44].

Alternatively, the extension of inflammation from the epithelial layer to deeper levels was detected in some highly responsive fish, such as the muscular layers of the intestinal wall. This was evident from a high lymphocyte number and rodlet cells/eosinophils with intense degranulation. The responses of these cells to protect the host from pathogens are commonly observed in the submucosal layer [45]. Teleost granulocytes release various molecules on activation by intracellular pathogens, such as peroxidase enzymes, reactive oxygen, antimicrobial peptides, and nitrogen intermediates. This causes persistent inflammation [46, 47]. The impairment of gut function, normal immune regulation, and gut microbiota may drive excessive inflammation through increased susceptibility of the gut to other pathogens [48]. Nevertheless, evidence of the pathophysiology mechanism in young and adults remains insufficient. Here, the relationship between mucosal pathology and inflammation due to coccidiosis was seen in our study. The highest intestinal epithelial necrosis and sloughing associated with intense inflammation caused by heavy infection. These severe infections aggravate epithelial injuries in the fishes and trigger epithelial cell desquamation and necrosis through direct cell burst from oocyst release and the inflammatory response [17, 39]. Similar responses to the previous publications on coccidia infection in various fishes were reported [16, 17, 28, 37]. Severe inflammation and mucosal damage are the lethal injuries that cause massive infected fingerling death.

## Conclusion

The novelty pathological changes accompanying coccidian infection in Asian sea bass fingerlings predominantly increase at the anterior intestinal area. These are severe mucosal inflammation extending to the deeper muscular layer, severe villi denudation, and mucosal necrosis. This emphasized the importance of the effect of coccidia on Asian sea bass fingerlings’ health and increase fatality. This is essential for management practices and applications, including disease prevention and control systems, and anti-parasitic drug protocols in Asian sea bass aquaculture. However, related pathological changes and in-depth mechanisms of individual genera of intestinal coccidia need to be determined further.

## Authors’ Contributions

WS: Conceptualization. WS, PS, CT, KK, and KJ: Methodology. WS: Validation. WS, PS: Formal analysis. WS: Investigation. WS: Resources. WS: Writing – original draft preparation. WS, ST: Writing – review and editing. WS: Supervision and editing. All authors have read and agreed to the submitted version of the manuscript.
